# ULK1 Signaling in the Liver: Autophagy Dependent and Independent Actions

**DOI:** 10.3389/fcell.2022.836021

**Published:** 2022-02-18

**Authors:** Sangam Rajak, Sana Raza, Rohit Anthony Sinha

**Affiliations:** Department of Endocrinology, Sanjay Gandhi Postgraduate Institute of Medical Sciences, Lucknow, India

**Keywords:** ULK1, autophagy, liver, non-alcoholic fatty liver disease, hepatocellular carcinoma

## Abstract

Liver is the primary organ for energy metabolism and detoxification in the human body. Not surprisingly, a derangement in liver function leads to several metabolic diseases. Autophagy is a cellular process, which primarily deals with providing molecules for energy production, and maintains cellular health. Autophagy in the liver has been implicated in several hepatic metabolic processes, such as, lipolysis, glycogenolysis, and gluconeogenesis. Autophagy also provides protection against drugs and pathogens. Deregulation of autophagy is associated with the development of non-alcoholic fatty liver disease (NAFLD) acute-liver injury, and cancer. The process of autophagy is synchronized by the action of autophagy family genes or autophagy (*Atg*) genes that perform key functions at different steps. The uncoordinated-51-like kinases 1 (ULK1) is a proximal kinase member of the *Atg* family that plays a crucial role in autophagy. Interestingly, ULK1 actions on hepatic cells may also involve some autophagy-independent signaling. In this review, we provide a comprehensive update of ULK1 mediated hepatic action involving lipotoxicity, acute liver injury, cholesterol synthesis, and hepatocellular carcinoma, including both its autophagic and non-autophagic functions.

## Introduction

Liver is a metabolic hub with several functions including synthesis of proteins such as albumin, lipid and carbohydrate catabolism, hormone synthesis, and detoxification. The hepatic cells are enriched with lysosomes and autophagosomes, which are the two cellular organelles that play a key role in liver physiology (Ueno and Komatsu, 2017). Hepatic autophagy is highly sensitive to the circulating hormone and amino acid concentrations, and provides glucose, amino acids, and free fatty acids, under starvation, for the production and synthesis of new macromolecules. Autophagy begins with membrane biogenesis and initiation of a pre-autophagosomal structure by the action of uncoordinated-51-like kinases 1 (ULK1) kinase complex (Ueno and Komatsu, 2017; Zachari and Ganley, 2017). The ULK1 kinase complex is comprised of ULK1, ATG13, RB1-inducible coiled-coil protein 1 (RB1CC1, also known as FIP200) and ATG101 (Ueno and Komatsu, 2017; Zachari and Ganley, 2017). The subsequent nucleation of the isolation membrane requires the class III PI3K complex (Ueno and Komatsu, 2017; Zachari and Ganley, 2017). The ATG12 and microtubule-associated protein 1A/1B-light chain 3 (LC3) conjugation systems play a critical role in the elongation and enclosure of the isolation membrane. The adaptor proteins such as SQSTM1/p62 possess a LC3-interacting region (LIR) that enables to bind LC3 and target proteins which have been ubiquitinated. After the engulfment of the cellular cargo, the autophagosomal membrane closes by the action of Syntaxin-17 (STX17) and then fuses with the help of the soluble N-ethylmaleimide-sensitive factor activating protein receptor (SNARE) proteins, synaptosomal-associated protein 29 (SNAP29) and vesicle-associated membrane protein 8 (VAMP8) assisted by the beclin 1-associated autophagy-related key regulator (also known as ATG14 or BARKOR), Pleckstrin homology domain containing family M member 1 (PLEKHM1) and Ras-related protein, Rab7 (Ueno and Komatsu, 2017; Zachari and Ganley, 2017). Autophagy derangement is associated with certain pathological symptoms and liver disorders, including fatty liver disease, viral hepatitis, acute liver injury, and hepatocellular carcinoma (HCC) (Ueno and Komatsu, 2017). The autophagy-related processes are coordinated by the *Atg* genes that encode the ATG proteins. Of these *Atg* genes, ULK1 which is the proximal gene responsible for the initiation of autophagy, has recently gained interest owing to its druggability and non-classical autophagy-independent actions (Alers et al., 2012; Papinski and Kraft, 2016; Saleiro et al., 2016; Nazio and Cecconi, 2017; Wang and Kundu, 2017). This review aims to recapitulate our present knowledge of ULK1 canonical and non-canonical signaling across different aspects of liver physiological and pathological processes.

## ULK1 Regulators and Targets

*Atg1*, a conserved serine/threonine kinase (Matsuura et al., 1997), is the only autophagy related kinase discovered in *S. cerevisiae*. The loss of *Atg1* in yeast results in early termination of autophagy (Matsuura et al., 1997). The *Atg1* homologues in mammals include uncoordinated-51-like kinases 1 and 2 (ULK1 and ULK2). ULK1 and ULK2 act in a redundant manner, and ULK1 deficiency causes a mild phenotype, in mice (Kundu et al., 2008). In mammals, ULK1 forms a multimeric complex binding to FIP200 (focal adhesion kinase family interacting protein of 200kD), ATG13 (autophagy-related 13), and ATG101 (autophagy-related 101) forming a complex termed as “autophagy initiation complex” or ULK1-ATG13-FIP200-ATG101 complex that initiates autophagy (Papinski and Kraft, 2016).

ULK1 is under direct control of the nutrient and energy sensors MTORC1 (mechanistic target of rapamycin complex 1) and AMP activated protein kinase (AMPK). Under nutrient rich environment, MTORC1 acts as a negative regulator of autophagy, and the starvation or rapalog mediated inhibition of MTORC1 elevates ULK1 kinase activity in the mammalian cells (Ravikumar et al., 2004). Studies show that both ULK1 and ATG13 of the initiation complex may be regulated through directed phosphorylation by MTORC1 (Hosokawa et al., 2009). ULK1 Ser757 phosphorylation by MTORC1 has been reported to inhibit its activity by interfering with its association with AMPK (Shang et al., 2011). In contrast to phosphorylation by MTORC1, AMPK mediated phosphorylation activates ULK1 to induce autophagy. The phosphorylation of Ser467, Ser555, Thr574, and Ser637 in ULK1 by AMPK during amino acid starvation, shows a direct link between AMPK and the ULK1 complex for the autophagic degradation of mitochondria (mitophagy) (Egan et al., 2011). The phosphorylation of ULK1 Ser555 by AMPK seems to regulate its mitochondrial translocation in hypoxia-induced mitophagy (Tian et al., 2015).

ULK1 regulates autophagy via direct phosphorylation of the key regulators of mammalian autophagy machinery. First, it phosphorylates its binding partners ATG13, FIP200, and ATG101 (Papinski and Kraft, 2016). ULK1 also phosphorylates many key members of the autophagy-specific PI3KC3 complex 1: Beclin-1, Vps34/PIK3C3 (vacuolar protein sorting 34/phosphatidylinositol 3-kinase catalytic subunit type 3), and AMBRA (Papinski and Kraft, 2016). Similarly, the mitophagy adaptor FUN14 domain containing 1 (FUNDC1) phosphorylation by ULK1 is essential for the coherent binding of LC3 to FUNDC1 and the progression of mitophagy (Wu et al., 2014). Additionally, ULK1 also invokes a reciprocal regulation of MTORC1 complex via an inhibitory phosphorylation of its subunit, Raptor, thereby sustaining autophagic flux (Dunlop et al., 2011). However, a negative feedback loop with AMPK phosphorylation also exists in context to ULK1 regulated autophagy (Loffler et al., 2011).

Intriguingly, several non-autophagic roles of ULK1 have been described recently, including its role in interferon signaling, ER-to–Golgi cargo transport, glycolysis, and immune response (Konno et al., 2013; Joo et al., 2016; Joshi et al., 2016; Li et al., 2016). Additionally, the regulation of the transcriptional activity of nuclear receptors involved in hepatic lipid metabolism by the autophagy-independent action of ULK1, has also been documented (Sinha et al., 2017; Rajak et al., 2020a). Owing to the pivotal role of ULK1 in both autophagy and non-autophagic processes (Wang and Kundu, 2017), along with its draggability, new ULK1 inhibitors have been recently developed (Egan et al., 2015).

## ULK1 and Hepatic “Mitophagy”

**Figure 1:**
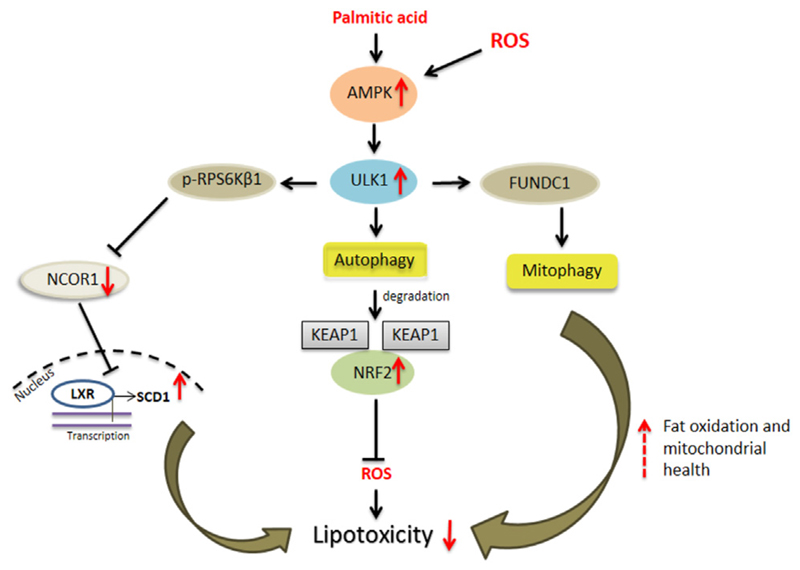
ULK1 and hepatic lipotoxicity. Uncoordinated-51-like kinases 1 (ULK1) *via* both autophagic and non-autophagic mechanisms limit cytotoxicity induced by saturated fats and oxidative stress in hepatocytes. The autophagy dependent mechanisms include selective degradation of damaged mitochondria or “mitophagy” and secondly by degradation of kelch like ECH associated protein 1 (KEAP1) thereby activating nuclear factor erythroid 2 like factor 2 (NRF2) anti-oxidant response. A non-autophagic pathway includes inhibition of ribosomal protein S6 kinase, polypeptide 1 (RPS6KB1)-nuclear receptor co-repressor 1 (NCOR1) signaling and thereby relieving liver X receptors (LXRs) mediated stearoyl-coenzyme A desaturase 1 (SCD1) transcription leading to conversion of saturated fats to their unsaturated species.

The liver was the organ where the process of autophagy was first described by Christian De duve, in 1960s (Appelmans et al., 1955). Although autophagy was originally designated as a non-selective and bulk degradative system that releases macromolecules under starvation, it is now well recognized as a highly selective process (Ueno and Komatsu, 2017). This type of autophagy, termed as selective autophagy, is recognized by the shuttling of its degraded products into the highly spatiotemporally regulated metabolic pathways of the liver. This process of selective autophagy has a role in degrading specific hepatic macromolecules including glycogen, lipids, proteins, and intracellular organelles such as mitochondria (Ueno and Komatsu, 2017). In the liver cells, the mitochondria play a key role in fat oxidation, and in the process, generate reactive oxygen species (ROS). The excessive ROS production in mitochondria may lead to mitochondrial damage resulting in hepatocyte injury. Therefore, maintaining mitochondrial health is crucial to sustain cellular metabolism and health. The AMPK driven activation of ULK1 and the consequent translocation of injured/fatigued mitochondria, results in mitophagic priming in the liver cells (Egan et al., 2011; Sinha et al., 2015; Tian et al., 2015) (Figure 1). This mechanism of ULK1 mediated mitophagy in liver is also associated with the direct phosphorylation of a mitophagy adaptor protein FUNDC1 (Wu et al., 2014; Singh et al., 2018). In this connection, thyroid hormone induced liver mitophagy but not bulk autophagy is regulated by ULK1, suggesting its exclusive role in selective vs. general autophagy. Furthermore, the silencing of ULK1 impaired mitophagy results in decreased thyroid hormone induced hepatic lipid oxidation (Sinha et al., 2015).

## ULK1 and Hepatic Lipotoxicity

Lipotoxicity is a term used to define tissue damage originating from the excessive accumulation of lipid species in the non-adipose tissues such as liver, pancreas, muscle and heart (Wasilewska and Lebensztejn, 2021). Lipotoxicity in the liver is associated with NAFLD, wherein excessive lipid accumulation drives liver inflammation, hepatocyte death, and fibrosis (Wasilewska and Lebensztejn, 2021). Autophagy plays a crucial role in controlling lipotoxicity during NAFLD, and the concurrent downregulation of the autophagy genes has been shown in advanced NAFLD, also termed as non-alcoholic steatohepatitis (NASH) (Czaja, 2016; Ramos et al., 2021). Autophagy protects against lipotoxicity by several mechanisms. First, autophagy is involved in the lysosome mediated degradation of intracellular lipid droplets within the hepatocytes. This selective autophagic process known as “lipophagy” is a key mechanism of action of several anti-steatosis agents (Sinha et al., 2014; Sinha et al., 2020a; Sinha et al., 2020b). Furthermore, autophagy may help to minimize injury in NASH by eliminating the damaged cell organelles or proteins which contribute to cellular dysfunction (Sinha and Yen, 2016). Autophagy defects increase the sensitization of the hepatocytes to endoplasmic and oxidative stress induced tissue damage and the induction of several proinflammatory cytokine expression from immune cells (Liu et al., 2015). Autophagy also helps in the prevention of the death receptor-mediated apoptosis from TNFα and Fas, which are involved in hepatocellular injury, in NASH (Amir et al., 2013).

ULK1, which is down-regulated in human NASH (Sinha et al., 2017), protects against hepatic lipotoxicity via both autophagy dependent and independent mechanisms (Figure 1). The activation of ULK1 has been implicated in hepatic lipophagy (Guha et al., 2019). Similarly, in a study by Park et al., the authors found that ULK1 prevented cellular lipotoxicity through the activation of NFE2L2 (Park et al., 2020). The NFE2L2/NRF2 (nuclear factor erythroid 2 like factor 2)-KEAP1 (kelch like ECH associated protein 1) pathway offers cytoprotection against cellular oxidative stress. Under normal conditions, KEAP1 suppresses NFE2L2 activation through its direct binding to the NFE2L2-CUL3-RBX1 complex, leading to NFE2L2 degradation (Ichimura and Komatsu, 2018). However, in absence of KEAP1, NFE2L2 is stabilized and enters the nucleus to activate the transcription of its target genes including NQO1 (NAD[P]H quinone dehydrogenase 1), GSTA1 (glutathione S-transferase alpha 1), and HMOX1/HO-1 (heme oxygenase 1) (Ichimura and Komatsu, 2018). SQSTM1/p62 is an autophagy receptor protein which activates the NFE2L2-KEAP1 pathway by specific binding to KEAP1, resulting in the stabilization of NFE2L2 (Ichimura and Komatsu, 2018). In this regard, ULK1 mediates the enhanced interactions between SQSTM1 and KEAP1 in the hepatocytes, which causes autophagic KEAP1 degradation and hence NFE2L2 activation (Lee et al., 2020; Park et al., 2020). Increased NEF2L2 levels are responsible for the induction of several anti-oxidant genes which protect against oxidative stress (Lee et al., 2020) (Figure 1). Furthermore, ULK1 is required for mitophagy and to enhance binding between SQSTM1 and PINK1 (PTEN induced kinase 1), in response to lipotoxicity (Park et al., 2020) (Figure 1). Apart from this autophagy-mediated mechanism, ULK1 also protects against lipotoxicity via a non-autophagic process (Sinha et al., 2017). ULK1 is known to phosphorylate and inactivate RPS6KB1 (ribosomal protein S6 kinase, polypeptide 1) that, in turn, regulates the nuclear entry of NCOR1 (nuclear receptor co-repressor 1), a nuclear corepressor. In the absence of ULK1 in hepatocytes, RPS6KB1 promotes the nuclear entry of NCOR1 which, in turn, inhibits the transcription of SCD1 (stearoyl-coenzyme A desaturase 1) by LXRs (Liver X receptors) (Sinha et al., 2017). SCD1 converts the lipotoxic saturated fatty acids into less toxic monounsaturated fats, therefore, the loss of SCD1 transcription due to low ULK1 expression leads to increased lipotoxic injury by saturated fats (Sinha et al., 2017) (Figure 1).

## ULK1 and Acute Liver Injury

Acute liver injury (ALI) refers to cellular damage due to the action of drugs and chemicals, characterized by oxidative stress, inflammation, and necrosis, and may often lead to liver failure (Hu et al., 2020). Drug-induced ALI commonly leads to mitochondrial damage and apoptosis. In humans, the intake of several drugs including antipyretics, antivirals, and chemotherapeutics, induces ALI (Hu et al., 2020). The overdose of acetaminophen (APAP), a commonly used antipyretic and analgesic, is reported to trigger ALI in mammals (Hu et al., 2020). The activation of autophagy appears to rescue hepatic injury induced by APAP (Hu et al., 2020). Rapamycin is known to significantly augment the autophagic process and decrease APAP-induced cell death in the cultured primary hepatocytes and mouse liver (Ni et al., 2012). Additionally, the induction of mitophagy protects against APAP induced mitochondrial damage in hepatocytes (Wang et al., 2019). Although the knockdown of autophagy-genes led to the aggravation of chemically induced liver injury, it exhibited a contrasting effect with respect to the genetic deletion of ULK1. In mice, the genetic silencing of autophagy gene *Atg7* worsened APAP-induced liver injury by the activation of caspases and c-Jun N-terminal kinase (JNK), resulting in the mitochondrial membrane depolarization, mitochondrial ROS accumulation, and hepatocyte apoptosis (Igusa et al., 2012). Interestingly, the *Ulk1/2* knockout displayed strong resistance to the APAP-induced ALI through the activation of JNK signaling in animals and *in vitro* (Sun et al., 2018). Mechanistically, the APAP-induced inhibition of MTORC1 activates ULK1 via decrease in its MTORC1 site phosphorylation. The activated ULK1 directly phosphorylates and enhances the kinase activity of MKK4/7 (mitogen-activated protein kinase 4 and 7), upstream kinases and the activator of JNK, to mediate the APAP-induced hepatic injury (Sun et al., 2018). These findings present an autophagy independent role of ULK1 in promoting APAP induced liver injury. However, ULK1 activation has been associated with protection against acute hepatic ischaemia-reperfusion (IR) injury, in an autophagy-dependent manner (Liu et al., 2019; Mohamed et al., 2021). Further studies using specific ULK1 inhibitors or genetic knock out models remain to be performed to evaluate the direct role of ULK1 in protecting against IR injury in liver.

## ULK1 and Cholesterol Biogenesis

**Figure 2:**
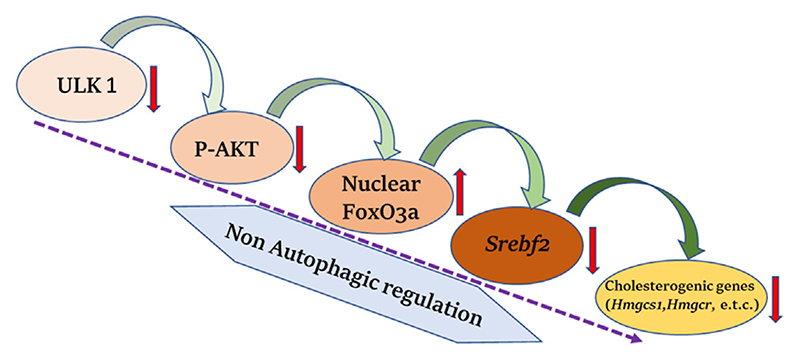
ULK1 and hepatic cholesterol biogenesis. Uncoordinated-51-like kinases 1 (ULK1) via a non-autophagic mechanisms regulates the biosynthesis of hepatic mevalonate/cholesterogenic genes. Loss of ULK1 leads to inhibition of protein kinase B (AKT) mediated Forkhead box class O 3a (FOXO3a) regulation in liver cells, wherein activated FoxO3a translocate to the nucleus and inhibits the transcription of sterol regulatory element binding factor 2 (*Srebf2*) required for the synthesis of cholesterol biogenesis genes.

In a recent study by Rajak et al., it was shown that ULK1 regulates the hepatic mevalonate (MVA) pathway via a non-autophagic mechanism (Rajak et al., 2020a). Cholesterol may be synthesized both endogenously *via* the MVA pathway in the liver or obtained from the food sources. Interestingly, the hepatic synthesis of cholesterol has been more closely linked to developing cardiovascular complications. The study unveiled a novel role of ULK1 signaling in regulating the expression of hepatic *de novo* cholesterol biosynthesis/mevalonate pathway genes, using an unbiased transcriptomics approach. The genetic silencing of *ULK1* in non-starved mouse (AML-12) and human (HepG2) hepatic cells as well as in the mouse liver, followed by transcriptomics analysis, uncovered a significant down-regulation of the genes involved in the mevalonate/cholesterol biosynthesis pathway, in the cells lacking ULK1. The loss of ULK1 caused impaired AKT activation, thereby, reducing the inhibitory phosphorylation of its target protein, FOXO3a, leading to its increased nuclear shuttling (Figure 2). Following its nuclear translocation, FOXO3a transcriptionally represses SREBF2/ SREBP2 (sterol regulatory element binding factor 2) expression. The decreased levels of SREBF2 further lead to the decreased expression of its target genes in the MVA pathway (Figure 2). This study identified ULK1 as a novel regulator of cholesterol biosynthesis and a druggable target to control cholesterol-associated pathologies, as an adjunct with the existing drugs such as statins.

## ULK1 and Hepatic Inflammatory Response

Autophagy plays an important role in regulating inflammation under various hepatic injury. Anti-inflammatory action of autophagy in liver macrophages, such as Kupffer cells, involves limiting inflammasome activation and release of interleukin 1 beta (IL-1β) cytokine (Lodder et al., 2015) associated with liver fibrosis. A recent study demonstrates that loss of ULK1 mediated autophagy in macrophages leads to increased inflammasome activation and pyroptosis (Shen et al., 2020). Interestingly, a non-autophagic contribution of ULK1 in repressing innate immune response is via phosphorylation of STING (Konno et al., 2013). The activation of the stimulator of interferon genes (STING) pathway in macrophages and non-parenchymal cell is responsible for increased cytokine production associated with NAFLD and may eventually lead to the development of HCC (Chen et al., 2021). Imbalance in the proportion of T helper cell 17 (Th17) and regulatory T cell (Treg) is associated with the progression of chronic liver disease (Drescher et al., 2020). In this context, autophagy has been shown to be an essential cell intrinsic process required to maintain functional integrity of Treg (Wei et al., 2016). Given the proximal role of ULK1 in the autophagic signaling, it may regulate hepatic inflammation by maintaining TH17/Treg balance, which needs to be further investigated. Therefore, targeting ULK1 signaling may be useful in countering the pro-inflammatory milieu often associated with liver diseases.

## ULK1 and Hepatic Cancers

Autophagy has a pleiotropic role in the initiation and establishment of HCC. The loss of *Atg* genes in the animal models, leads to the development of HCC via different mechanisms. The genetic loss of *Atg5* and *Atg7* increased the levels of SQSTM1/p62, oxidative stress, mitochondrial swelling, and genomic damage observed in the primary hepatocytes, and the deletion of the *p62* gene reduced the tumor size in *Atg7* deficient hepatic tumors (Umemura et al., 2016). The increased levels of p62, lead to the activation of Nrf2 that confers protection of the HCC-initiating cells from oxidative stress-induced cell death (Umemura et al., 2016). Notobaly, a high expression of p62 is observed in HCC tissues (Bao et al., 2014). Similarly, a higher incidence of spontaneous tumors, including HCC, was seen on the deletion of the autophagy regulatory gene *Beclin-1*, in mice (Yue et al., 2003). Mechanistically, Beclin-1 facilitates PP2A mediated degradation of c-Myc, leading to decreased cell division and cancer cell proliferation, indicating that *Beclin-1* acts as a haploinsufficiency tumor suppressor gene (TSG) in cancer (Cianfanelli et al., 2015).

Autophagy is known to enhance cancer cell survival and acts as a pro-survival and pro-metastatic process. Deregulated transcription of *Atg* genes is observed in HCC (Ji et al., 2019). Autophagy induction in HCC promotes cancer growth *via* induction of JNK/Bcl2 (Deng et al., 2018) and Wingless/Integrated (WNT) signaling (Fan et al., 2018). Autophagy is also implicated in the establishment of chemoresistance in response to the anti-HCC drug, Sorafenib. Furthermore, autophagy inhibitors sensitize HCC to sorafenib treatment (Shimizu et al., 2012; Zhao et al., 2021). Additionally, the expression of autophagy-related marker, LC3 has been linked to the poor outcomes in HCC patients with surgical resection (Lee et al., 2013).

**Figure 3:**
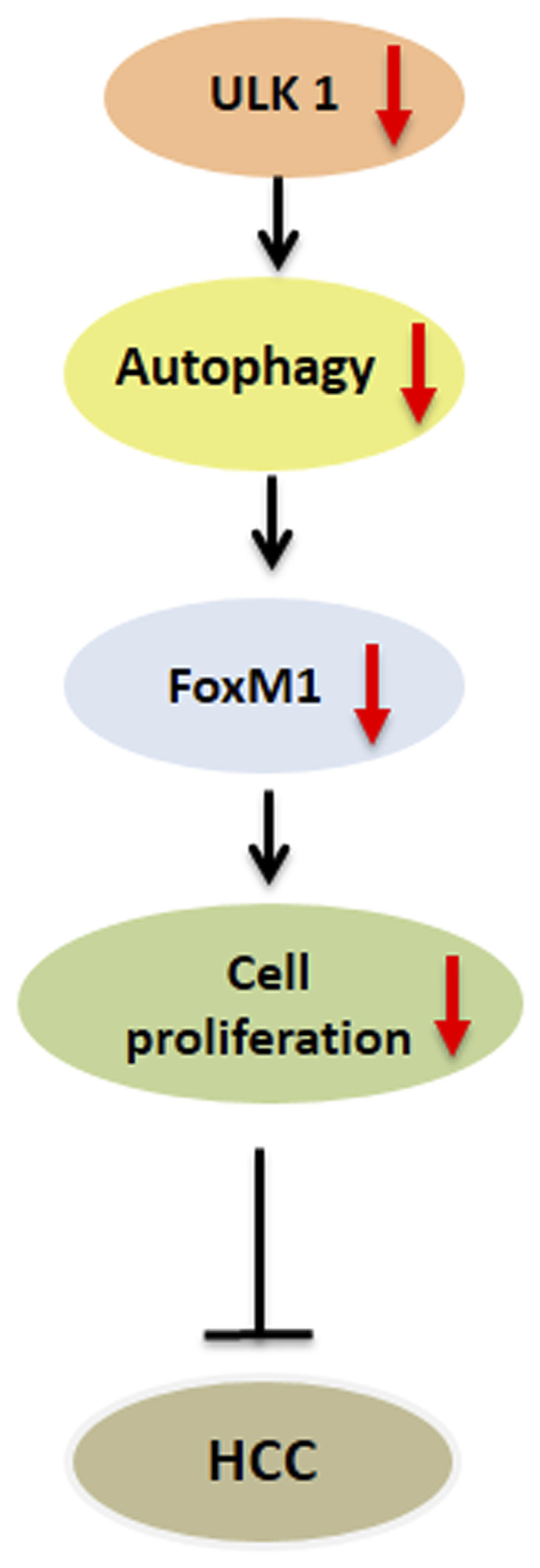
Role of ULK1 in hepatocellular carcinoma. Uncoordinated-51-like kinases 1 (ULK1)/autophagy inhibition reduces the expression of forkhead box protein M1 (FOXM1) oncogene and its downstream target genes regulating cell cycle progression in human hepatoma cells thereby limiting hepatocellular carcinoma (HCC) growth.

Besides LC3, ULK1 has also been shown as an important prognostic marker in HCC (Xu et al., 2013; Wu et al., 2018). ULK1 expression in paired HCC and para-cancerous tissues displayed a significant association with the tumor size after sex, age, histologic grade, cirrhosis and tumor, nodes, and metastases (TNM) adjustment (Xu et al., 2013). Upon survival analysis, the patients with high ULK1 expression showed worse survival time compared to those with low ULK1 expression (Xu et al., 2013). Additionally, both the genetic and pharmacological inhibition of ULK1 led to the inhibition of the proliferation and invasion of human HCC cells, and *Ulk1* deletion abrogated the tumor growth in a xenograft mouse model (Xue et al., 2020). Furthermore, the inhibition of ULK1, in combination with sorafenib, significantly suppressed the HCC progression as compared with sorafenib alone or vehicle treatment alone (Xue et al., 2020). Similarly, ULK1 was also found to regulate the expression of the oncogenic factor, FOXM1 by an autophagy dependent mechanism in HCC cell lines (Figure 3). FOXM1 promotes the growth of human HCC by its action on the transcription of genes related to proliferation, chemoresistance and metastasis (Figure 3). Inhibition of ULK1 by using siRNA or pharmacological inhibitors significantly down-regulated FOXM1 and its target gene transcription in HCC cell line (Rajak et al., 2020b) (Figure 3). Furthermore, a combinatorial administration of ULK1 and FOXM1 inhibitor synergistically decreased HCC proliferation (Rajak et al., 2020b). In summary, these studies unveil ULK1 as a novel therapeutic target for HCC (Liu et al., 2020) and indicate that targeting ULK1, in combination with other anti-cancer therapies, may be a promising interventional strategy for the treatment of HCC (Wang et al., 2018).

## Summary and Conclusion

**Figure 4:**
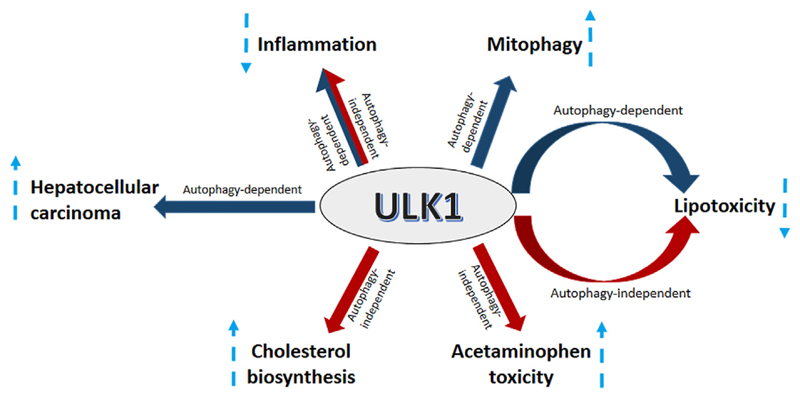
ULK1 signaling in the liver. Uncoordinated-51-like kinases 1 (ULK1) regulates several aspects of liver physiology and pathophysiology. ULK1 increases hepatic mitochondrial activity and lipid metabolism via induction of mitophagy. Additionally, ULK1 also supports HCC growth by regulating the expression of forkhead box protein M1 (FOXM1). ULK1 also mitigates lipid induced lipotoxicity and inflammation via both autophagy and non-autophagy mechanisms. Furthermore, ULK1 also increases acetaminophen (APAP) toxicity and hepatic cholesterol synthesis via a non-canonical autophagy independent signaling.

ULK1 serves as a critical regulator of autophagy needed to regulate divergent yet interconnected metabolic processes executed by the hepatic cells (Figure 4). However, not all actions of ULK1 lie within the bonafide domain of autophagy, suggesting that it may not be an exclusively autophagy-related gene, but in fact a kinase, which can also mediate non-autophagic functions (Figure 4). Finally, given the implications of ULK1 in several liver associated pathologies and its attribute to be a druggable kinase, future studies need to be directed to find specific and clinically relevant pharmacological modulators of ULK1 for the treatment of hepatic pathologies in humans.
